# Neuroinflammation and iron metabolism after intracerebral hemorrhage: a glial cell perspective

**DOI:** 10.3389/fneur.2024.1510039

**Published:** 2025-01-15

**Authors:** Jia-Jun Ju, Li-Hua Hang

**Affiliations:** ^1^Gusu School, Nanjing Medical University, The First People’s Hospital of Kunshan, Kunshan, China; ^2^Kunshan Cancer Pain Prevention and Treatment Key Laboratory, Kunshan, China

**Keywords:** intracerebral hemorrhage, neuroinflammation, iron metabolism, microglia, astrocytes, oligodendrocytes

## Abstract

Intracerebral hemorrhage (ICH) is the most common subtype of hemorrhagic stroke causing significant morbidity and mortality. Previously clinical treatments for ICH have largely been based on a single pathophysiological perspective, and there remains a lack of curative interventions. Following the rupture of cerebral blood vessels, blood metabolites activate resident immune cells such as microglia and astrocytes, and infiltrate peripheral immune cells, leading to the release of a series of inflammatory mediators. Degradation of hemoglobin produces large amounts of iron ions, leading to an imbalance of iron homeostasis and the production of large quantities of harmful hydroxyl radicals. Neuroinflammation and dysregulation of brain iron metabolism are both important pathophysiological changes in ICH, and both can exacerbate secondary brain injury. There is an inseparable relationship between brain iron metabolism disorder and activated glial cells after ICH. Glial cells participate in brain iron metabolism through various mechanisms; meanwhile, iron accumulation exacerbates neuroinflammation by activating inflammatory signaling pathways modulating the functions of inflammatory cells, and so on. This review aims to explore neuroinflammation from the perspective of iron metabolism, linking the complex pathophysiological changes, delving into the exploration of treatment approaches for ICH, and offering insights that could enhance clinical management strategies.

## 1 Introduction

Intracerebral hemorrhage (ICH) is the second most common type of stroke and a leading cause of death worldwide, with a mortality rate over 50% (1). Its incidence is projected to double by 2050 due to an aging population and increased anticoagulant use (2). Current treatment strategies such as hemostatic therapy, blood pressure control, and hematoma evacuation have limited clinical benefit due to their narrow focus on isolated aspects of ICH pathophysiology (3).

Neuroinflammation begins within minutes of blood vessel rupture and plays a key role throughout the stages of ICH, from the initial hemorrhage to brain recovery (4). The injury can be divided into primary and secondary stages. The primary injury occurs within hours due to hematoma compression, while secondary injury arises from toxic substances in the blood, disruption of the blood–brain barrier (BBB), and initiation of neuroinflammation (5). The lysis of erythrocytes and degradation of hemoglobin release iron ions after ICH, which contribute to lipid peroxidation, reactive oxygen species production, mitochondrial dysfunction, and neuroinflammation, leading to glial cell and neuronal damage (6). And the interplay between brain iron metabolism and glial cell function is largely dependent on inflammatory factors (7). Effective ICH treatment aims to maintain cerebral iron homeostasis, protect the BBB, and attenuation of secondary damage caused by neuroinflammation (8). This review focuses on the relationship between neuroinflammation and brain iron metabolism, summarizing the stages of neuroinflammation, the importance of iron homeostasis, and the regulatory pathways involved, providing insights for future treatment strategies.

## 2 Neuroinflammation after ICH

Neuroinflammation is an inflammatory cascade triggered by blood components, such as erythrocytes and their metabolites, thrombin, and fibrinogen. This response serves as a crucial defense mechanism but also exacerbates neurological damaget (9, 10). The process involves the activation of immune cells, increased expression and release of inflammatory cytokines, and heightened blood–brain barrier (BBB) permeability. Major immune cells involved include monocytes, macrophages, and neutrophils from peripheral sources, as well as resident brain cells like microglia and astrocytes (11). And Inflammatory mediators encompass cytokines (IL-1β, IL-6, TNF-α, IL-10), chemokines (CXCL1, CXCL8), matrix metalloproteinases (MMPs), prostanoids, complements, reactive oxygen species (ROS), inducible nitric oxide synthase (iNOS), blood-derived thrombin and plasmin, among others (12, 13).

The neuroimmune process can be categorized into four distinct stages:

### 2.1 Phase I: primary injury and initial inflammatory cascade reaction

Within the first 6 h after the occurrence of ICH, the hematoma expands and compresses the peripheral neurons, causing them to die (14). Simultaneously, the damaged vascular endothelium releases proteases, such as thrombin and fibrinogen, to trigger the coagulation cascade, and these proteases can directly bind to protease-activated receptors (PARs), triggering an inflammatory effect (15). Studies have indicated that PARs are expressed in neurons, microglia, astrocytes, and oligodendrocytes, with the activation timeline of PARs coinciding with the activation and polarization of microglia (16).

### 2.2 Phase II: glial cell activation and BBB remodeling

Microglia, with the P2RY12 receptor (a G protein-coupled receptor activated by ADP/ATP) respond rapidly to neuronal damage. Chemotactic microglia migrate to the injury site, releasing inflammatory factors and oxidative metabolites like IL-6, IL-1β, and TNF-α, which compromise tissue stability and barrier integrity (17, 18).

### 2.3 Phase III: peripheral circulating leukocyte recruitment

Within 12 h of ICH, M1 microglia produce chemokines such as CXCL2 and CCL2, attracting peripheral immune cells. Notably, natural killer (NK) cells, macrophages, and neutrophils increase significantly around the hematoma, with NK cells outnumbering other immune cells. Activated NK cells, through degranulation and chemokine production, exhibit proinflammatory characteristics and cytotoxicity, contributing to blood–brain barrier disruption and brain edema (19, 20).

### 2.4 Phase IV: damage repair and neurological recovery

Approximately 72 h after ICH, the inflammatory response gradually shifts to an anti-inflammatory phase. Over the next 2 weeks, M2 microglia with an anti-inflammatory phenotype predominate, releasing cytokines like IL-4, IL-10, and TGF-β, which facilitate hematoma resolution, tissue repair, and inflammation reduction (5, 21).

The neuroimmune response to ICH is dynamic and complex, initially focusing on injury control and damage mitigation through coagulation and inflammation. This phase, however, may lead to additional tissue damage due to inflammation and immune cell infiltration. As recovery progresses, the focus shifts from pro-inflammatory to anti-inflammatory activities. Understanding these phases and their mechanisms is crucial for identifying therapeutic targets to modulate inflammation, enhance repair, and improve neurological outcomes.

## 3 Brain iron metabolism and homeostasis

### 3.1 Physiological role of iron

Iron is one of the most abundant essential trace elements in the human body and plays a widespread role in various metabolic processes. As a vital component of several cytokines and cofactors, including heme groups and iron–sulfur clusters, iron is integral to electron transfer, oxygen transport, oxidative phosphorylation, as well as ATP and DNA synthesis (22). Iron exists primarily in two oxidation states—ferric iron (Fe^3+^) and ferrous iron (Fe^2+^)—which exhibit distinct biological activities and toxicities, playing critical roles in brain injury after ICH (23, 24). Fe^3+^, a less reactive oxidized state, is typically transported in the blood bound to transferrin. It is reduced to Fe^2+^ within cells via STEAP proteins to facilitate cellular utilization. However, when ferritin storage capacity is exceeded, excessive Fe^3+^ can lead to iron overload, indirectly triggering oxidative stress (25). In contrast, Fe^2+^ is highly reactive and participates in the Fenton reaction, generating hydroxyl radicals (·OH), which are among the most harmful reactive oxygen species. These hydroxyl radicals induce oxidative stress, resulting in lipid peroxidation, DNA damage, and protein oxidation, ultimately compromising the structural and functional integrity of neurons and glial cells (26, 27). The breakdown of hemoglobin during ICH releases Fe^2+^ into the peri-hematomal region, further amplifying oxidative damage and making Fe^2+^ a key driver of secondary brain injury in ICH.

### 3.2 Importance of brain iron homeostasis

Given the brain’s sensitivity to fluctuations in iron levels and the dual role in supporting critical biochemical processes, maintaining iron homeostasis within the brain is essential for normal neurological function. Under normal physiological conditions, iron serves as a cofactor for various enzymes involved in energy production, DNA synthesis, and neurotransmitter metabolism. It is also essential for myelin synthesis, which ensures efficient nerve signal transmission (28–30). Interestingly, the iron content in the brain increases with age, with the basal ganglia serving as the primary site of iron storage, making it particularly susceptible to iron accumulation (31). Misregulation of iron homeostasis can contribute to neurodevelopmental and neurodegenerative diseases through various mechanisms including Parkinson’s disease (PD) (32), Alzheimer’s disease (AD) (33), and Huntington’s disease (HD) (34). Therefore, the regulation of iron metabolism is imperative (35).

After ICH, red blood cell lysis releases hemoglobin, which is subsequently degraded, resulting in the accumulation of free iron, particularly ferrous iron (Fe^2+^) which triggers ferroptosis, an iron-dependent form of regulated cell death characterized by excessive lipid peroxidation and membrane disruption (6, 36). The excessive production of reactive oxygen species (ROS) during ferroptosis induces oxidative damage to lipids, proteins, and DNA, while mitochondrial depolarization and structural collapse impair energy metabolism, exacerbating neuronal death (37). Additionally, ferroptotic cells release debris and damage-associated molecular patterns (DAMPs), which activate glial cells such as microglia and astrocytes which secrete pro-inflammatory cytokines, including IL-1β, TNF-α, and IL-6, amplifying neuroinflammation (38). This cascade of oxidative stress, glial activation, and inflammation perpetuates a vicious cycle of neuronal loss, disruption of neural circuits, and significant structural brain damage. Moreover, blood–brain barrier disruption exacerbates brain edema and intracranial pressure, further aggravating neurological deficits (39–41).

### 3.3 Imbalance of brain iron homeostasis after ICH

**Figure 1:**
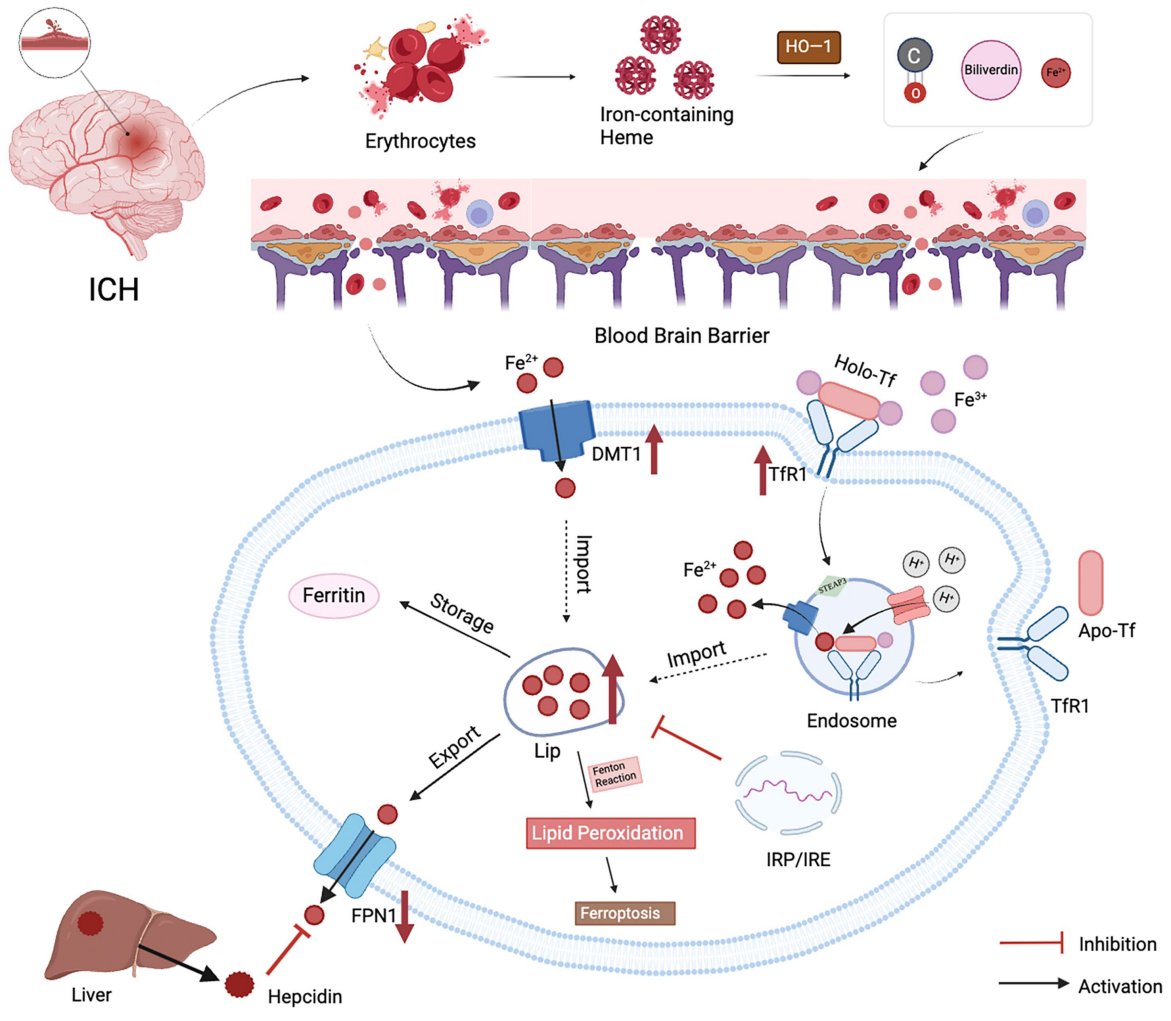
Possible causes of brain iron metabolism disorders after ICH. (1) When blood vessels rupture, damaged red blood cells release hemoglobin, which is degraded by HO−1 into iron ions, bilirubin, and carbon monoxide; (2) Progressive damage to the BBB leads to increased permeability and impaired function, resulting in increased passage of peripheral iron across the BBB into the brain; (3)The maintenance of iron homeostasis in the brain is accomplished through six processes: transport, uptake, storage, utilization, redox cycling, and excretion. Brain iron uptake is regulated by TfR1 and DMT1. Extracellular Fe^2+^ can directly enter cells via DMT1, while Fe^3+^ binds to transferrin and enters cells via endocytosis. Generated Fe^3+^ is transported out of cells by FPN1, the sole pathway for iron efflux. Hepcidin, primarily secreted by the liver, binds to FPN1, promoting internalization to control peripheral iron levels. After ICH, upregulation of TfR1 and DMT1 and downregulation of FPN1 result in excessive iron entering the labile iron pool, with iron storage, detoxification, and release regulated by ferritin. Additionally, the Fenton reaction can induce lipid peroxidation and iron-induced neuronal toxicity.

Preclinical studies have shown that brain iron levels increase up to threefold after ICH in rats, with this elevated iron environment persisting for at least 1 month. Although the exact mechanisms underlying iron accumulation in the brain remain unclear, several factors may contribute (Figure 1).

#### 3.3.1 Hemoglobin degradation

In cerebrovascular disease, the rupture of blood vessels leads to erythrocyte destruction and subsequent hemolysis, releasing hemoglobin, which is further degraded into iron ions, bilirubin, and carbon monoxide by the enzyme heme oxygenase-1 (HO-1) (41). HO-1 expression peaks on the third day after ICH and remains elevated until the 28th day, indicating that hemoglobin catabolism persists in the brain following ICH, leading to the accumulation of substantial free iron ions (7). Additionally, in a rat model of ICH, hemoglobin gradually spreads from the hematoma center starting on the first day post-hemorrhage, and the expression of the hemoglobin scavenger receptor CD163 around the hematoma is significantly upregulated, further confirming that hemolysis-induced hemoglobin release is a key factor in brain iron accumulation after ICH (42).

#### 3.3.2 BBB disruption

The BBB is a critical component of the central nervous system (CNS), consisting primarily of brain microvessel endothelial cells (BMVECs) and their tight junction proteins (including claudins, occludin, and junctional adhesion molecules), forming the basic framework. Pericytes and astrocyte end-feet envelop the endothelial cells, interacting with neurons, glial cells, and smooth muscle cells to form the “neurovascular unit (NVU)” (43). An intact BBB maintains low permeability, stringently controlling the entry of toxins and metabolites into brain tissue, thereby preserving CNS homeostasis (5).

**Figure 2:**
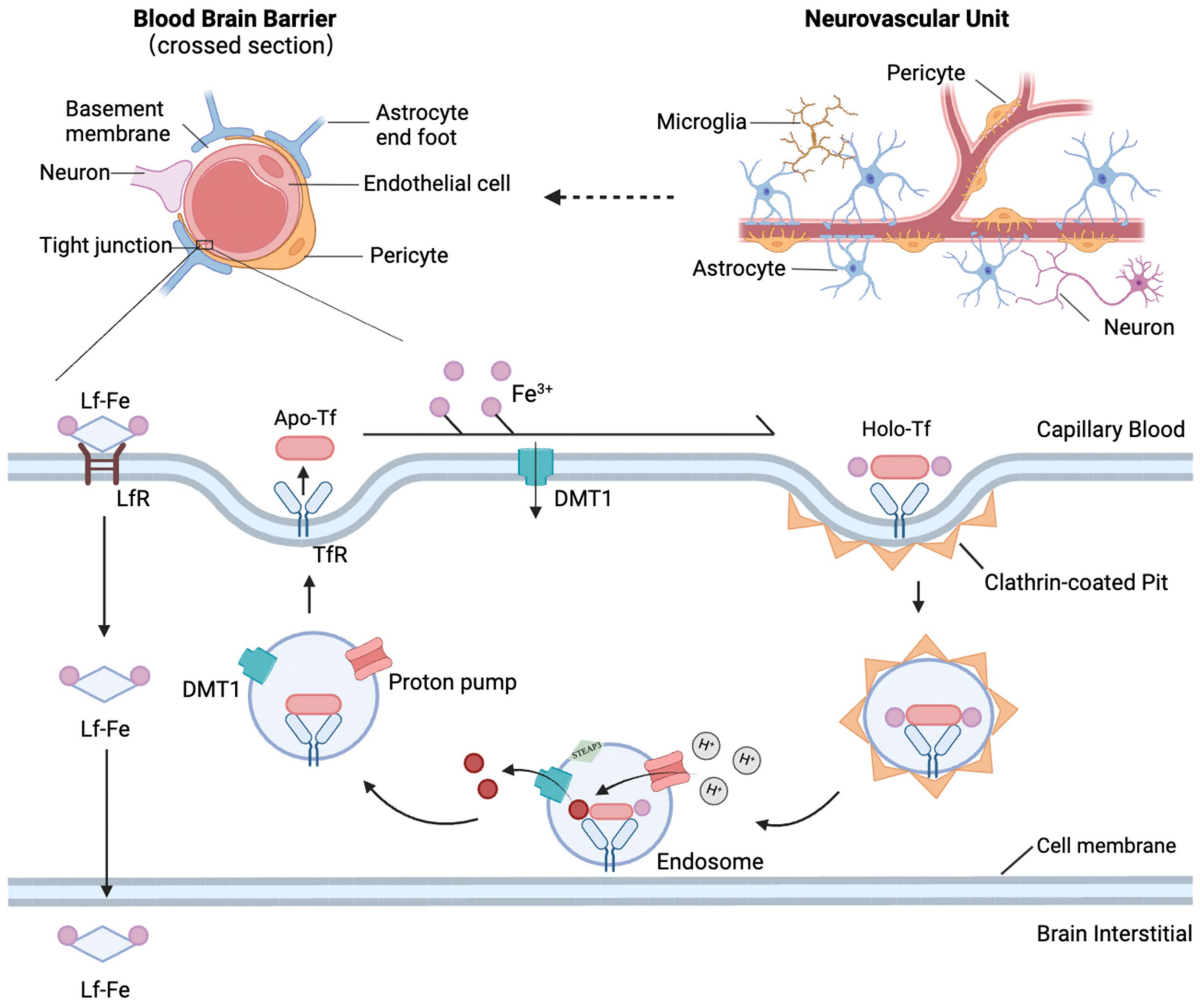
Structural diagram of the NVU and BBB and the main pathways of iron transport across the BBB. The NVU is composed of the BBB and neurons, with the BBB being a tightly functional unit consisting of microvascular endothelial cells, pericytes, basement membrane, and astrocyte endfeet. The mechanism of iron crossing the BBB involves the Tf/TfR pathway and the Lf/LfR pathway. The Tf/TfR pathway is likely the main route by which iron crosses the proximal luminal surface of BMVECs and involves binding, endocytosis, acidification and dissociation, translocation, and intracellular transport. Tf binds with the TfR1 receptor, forming an endocytic vesicle through TfR-mediated endocytosis. The h+ pump on the endosomal membrane acidifies the endosomal environment, causing Tf to dissociate from Fe^3+^, and STEAP3 reduces Fe^3+^ to Fe^2+^, which is then translocated across the endosomal membrane into the cytoplasm by DMT1. Released Fe^2+^ forms a complex with TfR1 and is returned to the cell surface via exocytosis. At the cell surface, TF dissociates from TfR1, becoming apo-transferrin, which then rebinds with extracellular Fe^3+^ to initiate the next iron transport cycle.

Under normal physiological conditions, iron ions traverse the BBB and are subsequently absorbed and utilized by brain cells. The process involves two transmembrane steps: first, iron crosses the luminal membrane (blood side) of BMVECs as Fe^3+^; then, as Fe^2+^, it crosses the abluminal membrane (brain side) and enters the brain parenchyma. The transferrin (Tf)/transferrin receptor (TfR) pathway is the primary route for iron to cross the luminal membrane, involving several steps: binding, endocytosis, acidification, dissociation, translocation, and intracellular transport (Figure 2). Briefly, Tf binds two Fe^3+^ ions to form a Tf-Fe^3+^ complex, which binds to TfR on the endothelial cell membrane, forming an endocytic vesicle mediated by TfR. H^+^-ATPase on the endocytic vesicle membrane acidifies the vesicle’s internal environment, lowering the pH to approximately 5.5–6.5, triggering the reduction of the Tf-TfR complex and the release of Fe^2+^. Fe^2+^ is then translocated across the endosome membrane into the endothelial cytoplasm via the divalent metal transporter (DMT1), while the majority of apo-Tf-TfR complexes are exocytosed back to the luminal membrane surface of endothelial cells, where they rebind extracellular Fe^3+^, initiating another cycle of iron transport (44). In addition to the Tf/TfR pathway, the lactoferrin (Lf)/lactoferrin receptor (LfR) pathway may also contribute to iron transport across the BBB (45) (Table 1).

**Table 1:** List of abbreviations and functions of proteins involved in iron metabolism (117).

	Abbreviation	Function
Transferrin/transferrin receptor	Tf/TfR	A major receptor pathway that mediates the transfer of Fe^3+^ ions into cells
Lactoferrin/lactoferrin receptor	Lf/LfR	A homologous protein of Tf and is a receptor pathway that mediates the transport of Fe^3+^ ions into cells
Iron regulatory protein/iron-responsive elements	IRE/IRP	Senses intracellular iron levels and upregulates the expression of iron metabolism-related proteins
Ceruloplasmin	Cp	A ferroxidase mediating the oxidation of Fe^2+^ ions to Fe^3+^ ions and promoting both iron release and iron uptake in brain cells
Hephaestin	Hp	A copper-containing protein homologous to Cp oxidizes Fe^2+^ ions to Fe^3+^ ions and assists in iron export
Hepcidin	Hepcidin	A circulating peptide that binds to FPN1, mediating degradation of FPN1
Ferritin	Ferritin	Ferritin-H has ferroxidase activity and is involved in the uptake and utilization of iron and Ferritin-L mainly plays a role in cellular iron storage
Divalent metal transporter 1	DMT1	Cellular iron importer responsible for ferrous iron uptake
Ferroportin1	FPN1	Cellular iron exporter responsible for ferrous iron release

However, structural damage to the BBB after ICH has been observed early in animal models. Studies have shown that within 12–48 h after ICH in rats, there is progressive damage to the BBB, leading to increased permeability and impaired function, resulting in an elevated influx of peripheral iron into the brain (46). Moreover, various blood components released after cerebral vessel rupture can enter the brain parenchyma through the compromised BBB, triggering oxidative stress and neuroinflammatory responses. This exacerbates BBB damage, worsening brain edema and neural injury following ICH, thus creating a vicious cycle (47).

#### 3.3.3 Abnormal expression of brain iron metabolism-related proteins

Abnormal expression of brain iron metabolism-related proteins may also contribute to iron accumulation in the brain. Under normal conditions, brain iron homeostasis is maintained through tightly regulated mechanisms of uptake, storage, utilization, and export, primarily involving transferrin, ferritin, and ferroportin (42). Fe^3+^ enters the brain via transferrin-mediated transport across the BBB, where transferrin-bound Fe^3+^ interacts with TfR1 on endothelial cells. Excess iron, in the form of non-transferrin-bound iron (NTBI), can bypass this pathway and enter cells via calcium channels or ZIP transporters (43). Ferritin, composed of heavy (H) and light (L) subunits, stores Fe^3+^ in a non-toxic form, preventing its participation in Fenton reactions that generate harmful ROS (44). Stored iron is mobilized through ferritinophagy, releasing Fe^2+^ for cellular functions (45). Iron export is mediated by ferroportin, the sole iron efflux protein, which is regulated by the hormone hepcidin. Elevated iron levels trigger hepcidin to bind ferroportin, causing its internalization and degradation, thus limiting iron release (46, 47). This dynamic system highlighting the critical roles of transferrin, ferritin, and ferroportin in maintaining brain iron balance.

Observations of brain tissue lesions in ICH rats revealed a significant increase in the expression of the iron transport proteins Tf, IRP-2, and DMT1, coupled with decreased expression of the iron export related protein FPN1 (48). Additionally, elevated levels of hepcidin, ferritin, and IL-6 were observed in ICH patients compared to controls, with these levels peaking on the 7th day after ICH and remaining higher than normal on the 14th day (49–51). These findings underscore the correlation between altered expression of iron metabolism-related proteins and brain iron accumulation after ICH.

## 4 Interplay between neuroinflammation and iron metabolism dysregulation after ICH

**Figure 3:**
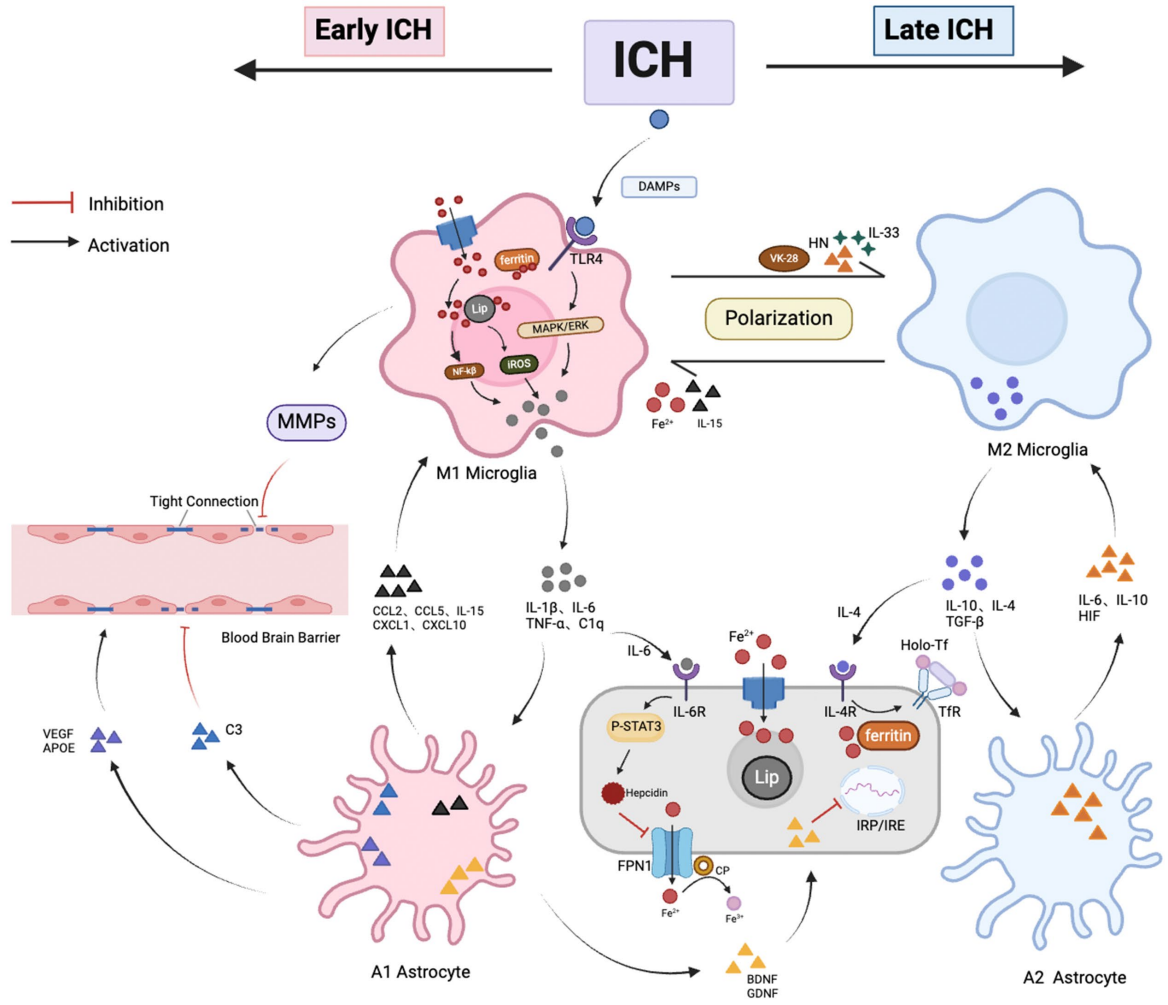
Crosstalk between glial cells and brain iron metabolism after ICH. (1) Interaction between Microglia and Astrocytes: After ICH, damaged neurons release DAMPs, triggering microglia to transition into the M1 phenotype, marking the onset of neuroinflammation. Activated microglia express inflammatory factors (such as TNF-α, IL-1β, C1q, and IL-6), stimulating A1 astrocytes, which increase levels of inflammatory and chemotactic factors, exacerbating neuronal damage. In the late stages of ICH, microglia gradually transition to the M2 phenotype, simultaneously releasing anti-inflammatory factors IL-4, IL-10, and TGF-β, which match receptors on A2 astrocytes. A2 astrocytes upregulate beneficial inflammatory factors (such as HIF, IL-6, IL-10), promoting neuronal recovery. The interaction between microglia and astrocytes is crucial for phenotype transition; (2) Relationship between microglial iron accumulation and neuroinflammation: After ICH, microglia encounter an elevated iron environment. This triggers the upregulation of DMT1 and ferritin expression, facilitating iron uptake via the NTBI pathway. Meanwhile, the anti-inflammatory cytokine IL-4, secreted by M2 microglia, upregulates TfR expression via the TBI pathway, influencing iron uptake by brain cells. Furthermore, microglia release IL-6, amplifying the expression of neuronal iron modulators, thus inhibiting FPN-mediated iron efflux. Additionally, microglia contribute to BBB breakdown by degrading TJ proteins through MMP release. Nevertheless, ferric ions overload or dilution, along with various iron chelators, can impact microglial polarization; (3) Relationship between iron accumulation in astrocytes and neuroinflammation: Activated astrocytes secrete BDNF and GDNF, which inhibit IRP and downregulate the expression of DMT1. In addition, astrocytes have a dual regulatory role on the BBB; they stimulate tight junctions by secreting VEGF, APOE, and other factors, but the release of the C3 complement leads to a decrease in junctional proteins, causing barrier imbalance. Astrocytes promote the coupling of iron with FPN1 by expressing CP, where internal Fe^2+^ may be transported out through FPN1 and oxidized to Fe^3+^, then bind to Tf and be taken up by brain cells. Additionally, astrocytes secrete HN, IL-15, and IL-33, which influence the polarization state of microglia.

Under normal physiological conditions, glial cells and brain iron homeostasis are essential for maintaining the normal functioning of the nervous system. There is a close interplay between neuroinflammation and iron metabolism disorders after ICH (Figure 3). First, both processes are critical pathophysiological changes following ICH, acting as key contributors to neurotoxicity and working as “co-conspirators” to exacerbate secondary brain injury. Second, there is a bidirectional relationship between these processes. Activated glial cells participate in the transport and storage of iron after ICH, leading to its accumulation in the brain. Excess iron, in turn, triggers neuroinflammatory responses by producing free radicals, which are involved in activating inflammatory signaling pathways and modulating the functions of inflammatory cells. Finally, iron chelators can form inactive complexes with iron ions, which have been shown to reduce free iron in the brain, inhibit the release of inflammatory factors, and thereby mitigate neuroinflammatory responses. In summary, inflammatory factors induce iron accumulation, leading to the generation of reactive oxygen species that worsen neural damage, while iron accumulation can, in turn, exacerbate neuroinflammatory responses.

### 4.1 Microglia

Microglia, the primary immune cells of the central nervous system (CNS), comprise 5–20% of the total cell population within the CNS. These cells serve as the first responders to CNS injury, playing a critical role in neuroinflammatory processes and acting as key markers of neuroinflammatory events (52, 53). After ICH, damaged neurons release DAMPs such as ATP, neurotransmitters, and nucleic acids, which subsequently activate microglia (16). Once activated, M1 microglia contribute to neuroinflammation via pathways including Toll-like receptor (TLR), nuclear factor-κB (NF-κB), and mitogen-activated protein kinase (MAPK) signaling. Conversely, M2 microglia activation is predominantly linked to signaling pathways such as Janus kinase/signal transducer and activator of transcription (JAK/STAT) and cyclic adenosine monophosphate (cAMP) (54, 55).

#### 4.1.1 Impact of microglia on brain iron metabolism after ICH

After ICH, activated microglia play a dual role by mediating neuroinflammatory responses and regulating brain iron metabolism. One key mechanism involves the induction of matrix metalloproteinases (MMPs), particularly MMP-2 and MMP-9, by activated microglia. These MMPs compromise the integrity of the BBB by degrading extracellular matrix components and tight junctions, thereby influencing brain iron homeostasis (56). Wu’s research indicates that MMP-9 mRNA expression significantly increases from 2 h to 5 days after ICH, with peak levels observed on the second day (57). Furthermore, treatment with 10% dimethyl sulfoxide (DMSO), a selective inhibitor of MMP-2 and MMP-9, has been shown to effectively reduce BBB permeability and mitigate ICH-induced edema in rats (58). These findings highlight the crucial role of microglia in the early stages of ICH, where their activation under neuroinflammatory conditions contributes to brain iron metabolism through the release of MMPs. Moreover, they suggest that MMPs may serve as a promising therapeutic target within the complex pathophysiological mechanisms of ICH.

Additionally, microglia express a range of iron metabolism-related proteins, including transferrin receptor (TfR), ferroportin-1 (FPN1), ferritin, divalent metal transporter 1 (DMT1), and hepcidin, which are integral to iron transport and storage (59). The expression of these iron metabolism-related proteins varies according to the microglial phenotype. M1 phenotype microglia, associated with pro-inflammatory responses, upregulate the expression of DMT1 and ferritin within the cytoplasm through the non-transferrin-bound iron (NTBI) pathway. In contrast, M2 phenotype microglia, which are linked to anti-inflammatory activities, release interleukin-4 (IL-4), an anti-inflammatory cytokine that enhances the expression of TfR. This upregulation of TfR facilitates iron uptake by brain cells via the transferrin-bound iron (TBI) pathway (60, 61).

Furthermore, microglia regulate brain iron metabolism through involvement in iron-related signaling pathways. Specifically, they modulate neuroimmune responses via the Toll-like receptor 4 (TLR4)-mediated signaling pathway, which significantly influences the pathological processes after ICH (62). Studies have demonstrated that TLR4 knockdown in ICH mouse models results in a marked reduction in the expression of inflammatory mediators such as IL-1β, IL-6, and TNF-α, underscoring the pivotal role of TLR4 in microglia-mediated inflammatory regulation (63). Further research has revealed that microglia release IL-6 to upregulate hepcidin expression, leading to the downregulation of FPN1. This downregulation restricts the efflux of iron from BMVECs, contributing to iron accumulation in the brain (64, 65). This process significantly impacts the dynamic homeostasis of iron and may play a crucial role in the brain’s repair mechanisms.

In summary, activated microglia play a critical role in regulating brain iron metabolism through multiple mechanisms after ICH. They contribute to the disruption of BBB integrity by inducing MMPs, which in turn impacts brain iron homeostasis. Additionally, microglia modulate iron transport and storage by expressing key iron metabolism-related proteins. Furthermore, microglia influence iron-related signaling pathways, particularly through the TLR4 mediated signaling pathway, which also regulates neuroimmune responses. These findings highlight the dual role of microglia in both neuroinflammatory responses and iron metabolism after ICH, suggesting that MMPs and TLR4 could serve as promising therapeutic targets. This underscores the importance of understanding the complex interactions between microglia, neuroimmune inflammation, and brain iron homeostasis.

#### 4.1.2 Impact of iron metabolism dysregulation on microglia and neuroimmune responses

Excessive iron accumulation in the brain significantly impacts microglial function and neuroimmune responses. Microglia play a critical role in mitigating iron overload by phagocytosing hemoglobin and necrotic tissues, a process essential for preventing secondary brain injury caused by iron toxicity (66). This mechanism not only helps maintain iron homeostasis within brain tissue but also reduces the neurotoxic effects of iron on the central nervous system. Moreover, the regulatory influence of iron ions on microglial polarization is notably complex. Studies have demonstrated that the bioactivity of iron and its interaction with cellular signaling pathways are key factors in determining microglial polarization states (60). The iron content within M1 microglia is generally higher than that in M2 microglia, which is associated with the exacerbation of inflammatory responses. Iron accumulation may promote the polarization of microglia toward the M1 phenotype, while reducing iron levels can induce M2 polarization, thereby helping to alleviate inflammation after ICH (67, 68). Moreover, iron accumulation generates large amounts of hydroxyl radicals through the Fenton or Harber-Weiss reactions, leading to the upregulation of NF-κB transcription. This, in turn, promotes the aggregation of TLRs and the release of proinflammatory cytokine TNF-α, which induces microglial polarization toward the M1 phenotype (69). This cascade plays a critical role in the inflammatory response and dysregulated iron metabolism after ICH.

NF-κB functions not only as a pivotal transcription factor in neuroimmune regulation but also as a crucial mediator in the crosstalk between iron metabolism and inflammatory responses. Its activation is positively correlated with the progression of cell death in ICH patients, underscoring a complex interplay between inflammation and neuroprotection post-brain injury. Dysregulation of iron metabolism exacerbates neuroimmune and inflammatory responses by increasing NF-κB expression, highlighting NF-κB as a potential therapeutic target. Although the inducible enzyme heme oxygenase-1 (HO-1) exhibits antioxidant, anti-inflammatory, and neuroprotective properties, its overexpression in microglia stimulated by lipopolysaccharide (LPS) leads to neurotoxic iron deposition. This deposition further promotes microglial polarization toward the M1 phenotype, accompanied by upregulation of inflammatory markers such as IL-1β, TNF-α, and caspase-1 (70, 71).

Furthermore, the use of iron chelators provides additional evidence for the impact of iron metabolism on glial cells and neuroinflammation. TNF-α, a key inflammatory mediator, induces brain cell necrosis after ICH through the activation of receptor-interacting protein kinase 1 (RIPK1). The iron chelator deferoxamine (DFO) has been shown to reduce the expression levels of TNF-α and RIPK1 in cerebral white matter, effectively mitigating cerebral white matter edema after ICH (72). Additionally, another iron chelator, VK-28, promotes microglial polarization toward the M2 phenotype, which accelerates hematoma clearance and facilitates neurological recovery (73). Thus, iron chelators play a crucial role in inhibiting iron accumulation, reducing neuroinflammatory responses, and minimizing secondary damage after ICH.

Dysregulation of iron metabolism profoundly affects microglial function and neuroimmune responses. And the iron-induced upregulation of NF-κB transcription is a key mechanism that drives neuroinflammation. Furthermore, iron chelators have been demonstrated to not only inhibit the progression of neuroinflammation but also minimize secondary brain injury, thereby promoting neurological recovery. These interconnected factors collectively play a critical role in the pathogenesis of ICH.

### 4.2 Astrocytes

Astrocytes, the most abundant cell type in the CNS, provide crucial trophic support to neurons, secrete a variety of cytokines including platelet-derived growth factors, glycine, cysteine, and TNF promote synapse formation and function, and interconnect with neurons and other glial cells through elongated protrusions that offer structural support to neurons (74). However, the function and behavior of astrocytes undergo significant alterations after ICH. In the early stages of ICH, M1 microglia are activated and induce A1-reactive astrocytes by secreting pro-inflammatory cytokines such as IL-6, IL-1β, TNF-α and complement component 1q (C1q), either individually or in combination. Upon activation, A1 astrocytes lose many of their normal functions and simultaneously secrete neurotoxins, which contribute to neuronal damage. These reactive astrocytes also collaborate with microglia to exacerbate neuroinflammation. Furthermore, astrocytes express cytokines such as IL-15, CCL2, CCL5, CXCL1, and CXCL10, which polarize microglia toward a pro-inflammatory phenotype (9). In the later stages of ICH, there is also communication between M2 microglia and A2 astrocytes. A2 astrocytes, once polarized, upregulate the expression of hypoxia-inducible factor (HIF), IL-6, and IL-10, thereby promoting neuronal repair and tissue recovery. When microglia clear necrotic material from the damaged area, the protrusions of A2 astrocytes occupy the void, facilitating tissue repair through scar formation (75, 76).

Furthermore, studies have shown that functional mitochondria released by astrocytes can be internalized by microglia, leading to the expression of humanin (HN), which promotes M2 microglial polarization and accelerates hematoma clearance (77). Additionally, astrocytes influence microglial polarization by secreting inflammatory mediators such as IL-15 and IL-33 (78). Thus, astrocytes and microglia play a crucial synergistic role in the neuroimmune response after ICH, acting as key regulators of inflammation and significantly impacting ICH prognosis by promoting tissue repair and neural remodeling.

#### 4.2.1 Impact of astrocytes on brain iron metabolism

Astrocytes collaborate with microglia to participate in neuroinflammation and play a crucial role in regulating brain iron homeostasis. They contribute not only to the physical composition of the BBB but also indirectly influence its stability and permeability through the secretion of various factors, thereby modulating iron transport between the vasculature and brain tissue. Consequently, astrocytes are vital for iron transport across the BBB and for maintaining iron homeostasis within the brain. The dense processes of astrocytes adhere tightly to the endothelial cells, enveloping them to form the “glial limiting membrane,” a charged basal membrane that ensures the integrity and proper functioning of the BBB (79). In inflammatory conditions, astrocytes can secrete vascular endothelial growth factor (VEGF) and apolipoprotein E (APOE), which enhance the expression of tight junction proteins and promote the development and maturation of the BBB (80). Specifically, APOE2 and APOE3 have been shown to inhibit inflammation and prevent the disruption of tight junctions in pericytes after ICH. Additionally, APOE3 binds to low-density lipoprotein receptor-related protein 1 (LRP1) on brain BMVECs, further stabilizing the BBB (81). Thus, astrocytes are indispensable for the regulation of iron transport between blood vessels and brain tissue, as well as for maintaining iron homeostasis within the brain.

Furthermore, the diverse functions of astrocytes in the nervous system underscore their pivotal role in regulating iron homeostasis and supporting neurotropism. Mature astrocytes also secrete angiotensinogen, which is converted into the effector molecule angiotensin-II upon binding to AT1 receptors on endothelial cells, thereby promoting the expression of tight junction proteins between these cells (82). The primary function of astrocytes is to facilitate iron transport to other glial cells or neurons rather than to store iron ions. Additionally, astrocytes produce neurotrophic factors such as brain-derived neurotrophic factor (BDNF) and glial cell line-derived neurotrophic factor (GDNF), which help prevent neuronal iron accumulation by inhibiting IRP activity associated with DMT1 expression (83).

Additionally, astrocytes regulate intracellular iron metabolism and transport through the ferroxidase activity of ceruloplasmin (CP). The ferroxidase activity of CP facilitates the coupling of iron with FPN1. Once Fe^2+^ is transported out of the cell by FPN1, it can be oxidized to Fe^3+^ by CP. Subsequently, Fe^3+^ binds to Tf, allowing for further uptake by brain cells (84). The significance of CP in neural iron metabolism is highlighted by studies in CP gene knockout mice, which exhibit abnormal iron accumulation in various brain regions, underscoring CP’s essential role in maintaining neural iron homeostasis (85). These findings underscore the complex regulatory role of astrocytes in neurological health and offer important insights into the mechanisms underlying abnormal iron metabolism in neurodegenerative diseases.

#### 4.2.2 Impact of iron metabolism dysregulation on astrocytes and neuroimmune responses

Astrocytes play a crucial role in regulating cerebral iron homeostasis and responding to neuroimmune challenges. Research has shown that astrocyte-derived hepcidin is essential for maintaining brain iron balance. In a mouse model with astrocyte-specific hepcidin knockout, a marked increase in FPN1 expression in BMVECs was observed, leading to a significant accumulation of iron within the brain parenchyma. Concurrently, astrocytes respond to elevated intracellular iron levels by increasing hepcidin secretion, which subsequently degrades FPN1 to limit the uptake of circulating iron by neuronal cells (86), These findings suggest that astrocytes exhibit a complex response to elevated intracellular iron, impacting both iron regulation and neuronal iron uptake. Moreover, astrocytes have been shown to respond to neuroinflammation and the presence of amyloid-beta (Aβ) proteins by upregulating ferritin expression, thereby modulating iron transport and metabolism. Iron chelators have been found to reduce brain iron levels by effectively inhibiting Aβ accumulation in astrocytes (81).

### 4.3 Oligodendrocytes

Oligodendrocytes are iron-rich cells within the brain that possess significant iron requirements and metabolic capacity, closely linked to their role in myelination metabolism (87), Due to their high sensitivity to iron levels, oligodendrocytes are particularly susceptible to damage and apoptosis in the context of iron accumulation after ICH. The elevated expression of the apoptosis-related protein caspase-3 in oligodendrocytes after internal capsule hemorrhage supports this observation (88). As key components of white matter, mature oligodendrocytes are vulnerable to injury as blood disperses along white matter tracts during hemorrhage, leading to atrophy and damage. Moreover, pro-inflammatory cytokines released by microglia and astrocytes following ICH directly induce oligodendrocyte apoptosis, thereby exacerbating white matter injury (89). Fortunately, oligodendrocytes retain the ability to regenerate and repair following CNS damage. During the acute phase of perihematomal white matter injury, oligodendrocyte precursor cells (OPCs) proliferate to generate new oligodendrocytes, which remyelinate denuded axons and restore neuronal function (90). Additionally, oligodendrocytes secrete the acute-phase response protein haptoglobin (Hp), which protects neuronal cells from damage caused by hemolytic products, including neurotoxic iron (91, 92). Shen et al. (93) discovered that under hemorrhagic conditions, OPCs undergo GPX4-dependent ferroptosis, which impedes the remyelination of damaged axons, representing a critical mechanism underlying white matter injury and neurological dysfunction. Meanwhile, the use of iron chelators has been shown to reduce cellular apoptosis and promote myelination, thereby offering significant protection against white matter injury and aiding in the recovery of neurological motor function.

Additionally, Wu’s research was the first to reveal the interaction between immune cells and OPC ferroptosis following cerebral hemorrhage. The inflammatory cytokine IL-10, released by microglia, can reduce the intracellular phospholipid content containing unsaturated fatty acids via the STAT3/DLK1/ACC pathway, thereby rescuing OPCs from ferroptosis induced by hemorrhage. This, in turn, ameliorates white matter injury and improves chronic neurological deficits (94). However, current understanding of the interaction between oligodendrocytes and iron metabolism remains limited. Further research is essential to explore whether oligodendrocytes act synergistically with other glial cells in neuroinflammation after ICH, and to elucidate their relationship with brain iron metabolism. Such insights may contribute to the development of new therapeutic strategies to mitigate neurological damage and enhance recovery.

## 5 Conclusion and outlook

This article elucidates the interactions between neuroinflammation and brain iron metabolism after ICH. Neuroinflammation arises from a combination of factors, including the mechanical injury caused by ICH, stimulation by blood components, apoptosis and necrosis of cells surrounding the hematoma, and activation of immune cells. Additionally, the degradation of hemoglobin, disruption of the BBB, and abnormal expression of iron metabolism-related proteins after ICH lead to disturbances in brain iron metabolism. The consequent increase in brain iron levels exacerbates iron deposition, which results in direct cytotoxicity and oxidative stress. The resulting vicious cycle between iron imbalance and neuroinflammatory processes ultimately worsens neurological damage. Therefore, elucidating and exploring the mechanistic pathways underlying this process is crucial for the development of effective ICH treatments.

Therapeutic strategies targeting iron homeostasis, such as deferoxamine (DFO), show potential for mitigating brain injury after ICH. DFO, a widely studied iron chelator, has demonstrated promise in preclinical models by reducing harmful iron accumulation and exhibiting neuroprotective effects, including anti-apoptotic, anti-oxidative, anti-inflammatory, and anti-phagocytic actions (95, 96). It also suppresses ROS production and blocks hemoglobin-induced glutamate neurotoxicity (97–99). However, despite encouraging early-phase trial results, phase II trials failed to show significant therapeutic benefits (100, 101). This may be attributed to suboptimal dosing regimens, reduced bioavailability due to differences in administration routes (102–104), and DFO’s limited ability to cross the BBB. Although BBB disruption in ICH may improve permeability, partial BBB integrity can still hinder its efficacy (73). Additionally, off-target effects, such as nephrotoxicity and systemic adverse reactions, especially with prolonged use, further challenge its clinical application (105–107).

Another promising avenue in mitigating ICH-induced brain injury involves lipid peroxidation, which is central to ferroptosis. The primary antioxidant pathways regulating ferroptosis include GPX4 and the FSP1–CoQ10–NAD(P)H axis. A recently identified alternative pathway, the GCH1–BH4–DHF axis, presents additional potential, though further investigation is required. Edaravone, a free radical scavenger, has shown effectiveness in alleviating oxidative damage by neutralizing hydroxyl radicals and peroxynitrite. This action reduces brain edema and activates the Nrf2/ARE signaling pathway, which enhances endogenous antioxidant defenses, increasing the expression of enzymes such as glutathione, superoxide dismutase (SOD), and catalase (108–110). While edaravone has been shown to alleviate neurological deficits, reduce hematoma volume, and improve activities of daily living in ICH patients, it does not significantly reduce mortality when administered within 7 days of ICH onset. Additionally, the high heterogeneity and generally low quality of existing clinical trials make it difficult to recommend edaravone as a routine treatment for acute ICH (111, 112). Thus, further high-quality studies are needed to clarify its therapeutic role.

Ferroptosis inhibitors are another promising class of therapeutics in the context of ICH. Preclinical studies have demonstrated that compounds like Ferrostatin-1 and Liproxstatin-1 can effectively reduce ferroptosis-related molecular markers, such as the downregulation of GPX4 and increased lipid peroxidation. These inhibitors also alleviate inflammation and oxidative stress, reduce neuronal damage and brain edema, and promote neurological recovery in rodent models of ICH (113–116). These encouraging findings highlight ferroptosis inhibition as a potential therapeutic target for ICH. However, the clinical application of ferroptosis inhibitors is still in its infancy, and their safety and efficacy in human trials have yet to be thoroughly validated. Ongoing research aims to develop more selective inhibitors that specifically target ferroptosis without disrupting normal cellular functions, a crucial step for advancing these therapies into clinical practice.

In conclusion, therapeutic strategies targeting iron homeostasis, including iron chelation, antioxidant treatment, and ferroptosis inhibition, present promising approaches to manage ICH-induced brain injury. However, significant challenges remain in translating these therapies from preclinical studies to effective human treatments. Future research should focus on elucidating the precise molecular mechanisms governing iron metabolism and neuroinflammation, as well as exploring how these pathways could serve as therapeutic targets in ICH. With continued investigation and refinement, these strategies hold the potential to significantly improve outcomes for ICH patients.
